# Clinical spectrum and survival analysis of 145 cases of HIV-negative Castleman’s disease: renal function is an important prognostic factor

**DOI:** 10.1038/srep23831

**Published:** 2016-03-31

**Authors:** Lu Zhang, Zhiyuan Li, Xinxin Cao, Jun Feng, Dingrong Zhong, Shujie Wang, Daobin Zhou, Jian Li

**Affiliations:** 1 Department of Hematology, Peking Union Medical College Hospital, Chinese Academy of Medical Sciences and Peking Union Medical College, Beijing, China; 2 Department of Pathology, Peking Union Medical College Hospital, Chinese Academy of Medical Sciences and Peking Union Medical College, Beijing, China

## Abstract

Castleman’s disease (CD) is a rare lymphoproliferative disorder with clinical features and prognostic factors that are incompletely characterized. This retrospective single-center study reviewed the largest HIV-negative CD patient cohort (n = 145) to date. By clinical classification, we identified 69 patients (47.6%) as unicentric CD (UCD) and 76 patients (52.4%) as multicentric CD (MCD). Pathological classification identified 74 patients (51.0%) with the hyaline-vascular variant, 51 patients (35.2%) with the plasma-cell variant, and 20 patients (13.8%) with a mixed variant. After a median follow-up duration of 58 months (range, 1–180 months), the 1-year and 5-year survival rates were 95.1% and 91.0%, respectively. UCD patients exhibited significantly better survival (1-year and 5-year survival rates of 98.5% and 97.1%, respectively) compared with MCD patients (1-year and 5-year survival rates of 92.1% and 85.5%, respectively; p = 0.005). By univariate and multivariate Cox regression analyses, the estimated glomerular filtration rate < 60 ml/min (with the MDRD equation; hazard ratio = 4.60; 95% confidence interval, 1.50–14.12; p = 0.008) was clinically significant and represented an independent predictor for death in MCD patients. In summary, this large-scale study suggests that UCD patients enjoy better survival than MCD patients and that renal function is an important prognostic factor for MCD patients.

Castleman’s disease (CD, angiofollicular lymph node hyperplasia) is a rare lymphoproliferative disorder that was first described in 1956 by Benjamin Castleman and colleagues^1^. As a highly heterogeneous entity, CD comprises at least two distinct diseases (unicentric CD, UCD, and multicentric CD, MCD) with different clinical characteristics and prognoses^2^. Because of the rarity of CD, knowledge about its clinical characteristics has accumulated slowly, and is mostly described in case reports^3^. Despite the publication of several larger case series since 2011^4^,^5^,^6^,^7^,^8^, few studies have focused on survival analysis or characterization of the prognostic factors for CD patients. Moreover, although human immunodeficiency virus (HIV) status is recognized to be an important factor in patients with CD^9^, previous studies have enrolled both HIV-positive and HIV-negative patients^3^,^4^ (as well as patients with an ambiguous HIV status^5^,^6^). HIV status itself might represent a risk factor for survival in CD patients^4^, it may be helpful to perform survival analysis in HIV-negative patients to identify risk factors other than HIV status for CD patients, especially in countries with a low HIV prevalence, such as China^10^. Herein, we report 145 HIV-negative CD patients with available clinical and pathological data; moreover, we attempted to delineate the survival pattern and to identify prognostic factors for survival in these patients.

## Patients and Methods

### Patients

This retrospective single-center study screened 151 patients who were diagnosed as CD at Peking Union Medical College Hospital (PUMCH) from January 2000 to June 2015. Pathological specimens were retrieved from the PUMCH pathology database and reviewed by two experienced pathologists. The diagnosis of CD was corrected in three patients and confirmed in 148 patients. Because patients with an underlying systemic inflammatory disease or malignancies that might have a CD etiology of that differed from that observed in most CD patients^9^. There were two patients with concomitant systemic lupus erythematosus and one patient with lung cancer who were excluded, resulting in a total of 145 CD patients who were eligible for this study. Demographic, clinical, laboratory, and treatment-related data, including age, sex, past medical history, symptoms at presentation, Eastern Cooperative Oncology Group (ECOG) status, physical examination, serological results, radiological findings, pathological results, and treatment strategies were extracted from patient medical records. All patients underwent computerized tomography (CT) scans or an ultrasound of the involved regions or superficial lymph nodes. All patients underwent HIV serology screening, which all yielded negative results. Clinical classifications were based on a physical examination, radiological findings, and surgical findings. UCD was defined as localized disease (one site of lymphadenopathy); while MCD was defined by the involvement of two or more lymph nodes or regions^5^,^6^.

This study was performed in accordance with relevant guidelines and regulations and was approved by the PUMCH Ethics Committee. Informed consent was obtained from our patients.

### Follow-up

Patient follow-up was conducted via interviews at an outpatient clinic, telephone contacts, letters, and analyses of information documented in the PUMCH databases. Patients were followed until May 31, 2015 and the survival status for each patient was documented. Survival time was defined as the period from diagnosis to either death or the last follow-up.

### Statistical Analysis

Statistical analyses were performed with SPSS statistical software (version 13.0; SPSS Inc., Chicago, IL, USA). The Kaplan–Meier method was used to depict survival curves and calculate survival rates at 1 and 5 years after the diagnosis of CD. The log-rank test was used to compare survival curves for UCD and MCD patients. Because MCD was considered to be a disease that was very distinct from UCD, both in its clinical course and underlying pathogenesis^2^,^9^, further analysis was performed for MCD patients to identify possible prognostic factors. To compare survivors and non-survivors, potential risk factors were first screened using chi-square tests for categorical covariates and *t*-tests for continuous covariates; p < 0.05 was used as a threshold for statistically significant differences. Univariate Cox regression was used to estimate hazard ratios (HRs) and to test survival differences between groups. Variables with p < 0.10 by univariate analysis were further analyzed by multivariate Cox regression. All clinically significant parameters (p < 0.05) were considered to represent independent predictors of survival.

## Results

### Patient Characteristics

Appendix 1 (see supplement) shows the clinical characteristics of our patients. A total of 145 HIV-negative CD patients were enrolled in this study, including 69 (47.6%) males and 76 (52.4%) females. The median age at diagnosis was 40 years (range, 11–79 years). Six patients (4.1%) had diabetes and 21 patients (14.5%) had hypertension. In our cohort, 69 patients (47.6%) were clinically classified as UCD and 76 patients (52.4%) were classified as MCD. There were 79 patients (54.5%) who had lesions that involved one side of the diaphragm. Pathologically, 74 patients (51.0%) were classified as hyaline-vascular (HV) variant, 51 patients (35.2%) were classified as plasma-cell (PC) variant, and 20 patients (13.8%) were classified as a mixed variant. There were 19 patients (13.1%) who had POEMS (polyneuropathy, organomegaly, endocrinopathy, monoclonal protein, skin changes) syndrome and seven patients (4.8%) who had paraneoplastic pemphigus (PNP). Notably, one patient (0.7%) fulfilled the diagnostic criteria of TAFRO (thrombocytopenia, anasarca, bone marrow fibrosis, renal dysfunction, organomegaly) syndrome. Finally, 17 patients (11.7%) had an estimated glomerular filtration rate (eGFR) < 60 ml/min (calculated using the Modification of Diet in Renal Disease equation^11^). In the 76 MCD patients, 15 patients (19.7%) had an eGFR < 60 ml/min.

### Treatment

There were seven patients (4.8%) who received no treatment other than biopsy. All UCD patients (n = 69) and 11 MCD patients (many with intra-abdominal lesions) received surgery. A total of 13 patients (9.0%) received steroids with or without intravenous immunoglobulin and immunosuppressive agents. Among 54 patients (37.2%) who received combination chemotherapy, 47 patients received CHOP (cyclophosphamide, doxorubicin, vincristine, and prednisone) or CHOP-like chemotherapy (eight had Rituximab-CHOP), and seven patients were administered high-dose dexamethasone based therapy (see Appendix 1). Additionally, eight patients (5.5%) received autologous stem cell transplantation (ASCT), which was a recommended first-line treatment for young POEMS patients with normal organ function^12^.

### Outcomes

**Figure 1:**
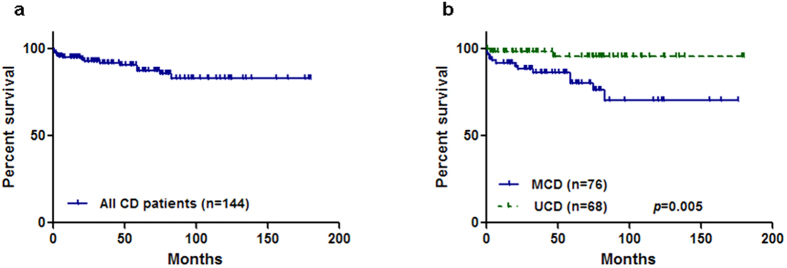
(**a**) Survival curve of all patients with Castleman’s disease. (**b**) Survival curves of multicentric Castleman’s disease versus. unicentric Castleman’s disease.

The median follow-up duration was 58 months (1–180 months), and one patient (an 11-year-old female) was lost to follow up. In the remaining 144 patients, 15 (10.4%) had died, 13 had MCD, and two had UCD. The 1-year and 5-year survival rates for all CD patients were 95.1% and 91.0%, respectively (Fig. 1a). For UCD and MCD patients, the 1-year and 5-year survival rates were 98.5% and 97.0%, and 92.1% and 85.5%, respectively. Using the log-rank test, UCD patients had significantly better survival (p = 0.005) compared with MCD patients (Fig. 1b), which validated the additional survival analyses that targeted MCD patients.

### Prognostic factors

In total, 76 MCD patients were eligible for further analysis. The clinical features of MCD patients are listed in Appendix 2 (see supplement), including risk factor distributions between survivors and non-survivors. Compared with survivors, non-survivors had a lower proportion of male patients (p = 0.03), higher rate of tuberculosis (TB) history (p = 0.03), higher rate of POEMS syndrome (p = 0.04), and lower eGFR (p = 0.009).

Based on univariate logistic regression (Table 1), three risk factors were found to be significant predictors for survival in MCD patients. A prior history of TB (HR = 4.51; 95% confidence interval (CI), 1.23–16.47; p = 0.02), the presence of POEMS syndrome (HR = 3.03; 95% CI, 1.01–9.04; p = 0.047), and eGFR < 60 ml/min (HR = 4.89; 95% CI, 1.63–14.73; p = 0.005) were each associated with poor survival.

**Figure 2:**
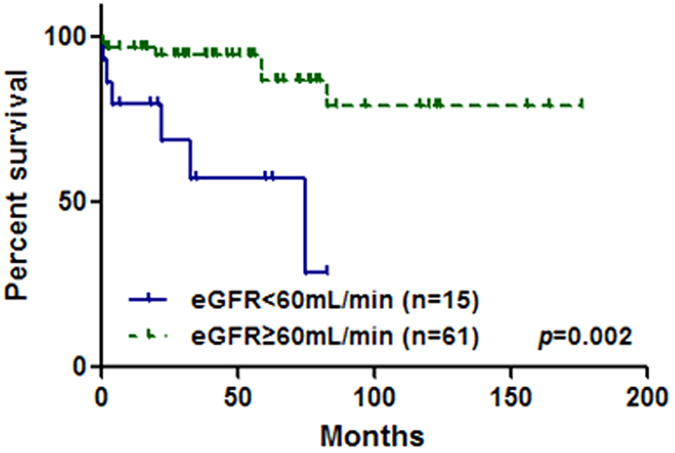
Survival curves of patients with eGFR ≥ 60 ml/min versus. <60 ml/min.

After incorporating variables with P < 0.10 in univariate analysis, multivariate Cox regression (Table 1) revealed that only eGFR < 60 ml/min (HR = 4.60; 95% CI, 1.50–14.12; p = 0.008) was a clinically significant and independent predictor for death in MCD patients (Fig. 2).

## Discussion

Before 2011, knowledge about CD was mainly based on case reports^3^. Since 2011, several case series have been published; however, the patient numbers in these previous studies were small^4^,^7^,^8^ and only two studies enrolled more than 100 cases (Table 2)^5^,^6^. Moreover, these two previous studies had several limitations.

First, they enrolled patients since the 1940s or 1970s, respectively, which might introduce considerable error, especially for retrospective studies. Moreover, HIV is an important factor in the pathogenesis of CD^9^, and it was not discovered until the 1980s. Similarly, the CT scan, a vital tool for assessing CD, was not available until the 1970s in the Western world and the 1990s in China.

Second, a recent study from China^6^ might have some hospital-based biases that may interfere with the interpretation of study results. As a large center that treats PNP^13^, that hospital in China collected many PNP patients, and those patients with concomitant CD were enrolled in their study^6^, resulting in a high frequency (32.4%) of PNP, which was not representative of typical CD cases. Indeed, in a large meta-analysis, the rate of PNP was 1.3%^3^; in another study that enrolled more than 100 patients, the rate was 1%^5^; in our study, it was 4.8%. As CD patients with PNP had different clinical characteristics (including clinical classifications and pathological distributions) and outcomes^6^, the results of one CD study that enrolled too many PNP patients might not reflect the general characteristics of CD disease.

Third, HIV status is vital for CD studies as it is both related to the pathogenesis of CD^9^ and imposes a strong influence on patient outcomes^4^; however, both studies had an ambiguous HIV status for their patients (Table 2), which might exert a ‘hidden’ impact on the results. To address these aforementioned limitations, we paid careful attention to the design of this current study. Therefore, we enrolled patients in the last 15 years whose medical records and pathological specimens were complete and available. Moreover, along with advances in modern medicine, we also witnessed an improvement in the prognosis of CD patients (Table 2). Additionally, as a large general hospital, we enrolled patients from different departments, resulting in a patient population that was representative of the general CD population, which was similar to those of Dispenzieri *et al.*^5^ for patient characteristics (see Table 2 for age, gender, and the UCD/MCD distribution, which differed from the population of Dong *et al.*^6^). Finally, as HIV screening was routine for our hospitalized patients during the past 15 years, unambiguous data on HIV status was available for our patients, which helped us to exclude the possible impact of HIV on survival analyses.

UCD is a localized disease that is sometimes associated with systemic symptoms. In contrast, MCD is a systemic disease that is often associated with generalized peripheral lymphadenopathy, hepatosplenomegaly, fevers, and night sweats. Unlike UCD, MCD is strongly associated with immunosuppression (e.g., HIV infection) and human herpesvirus 8 (HHV-8) infection^9^,^14^. Thus, MCD is considered to be a very distinct disease from UCD, both in its clinical course and underlying pathogenesis^2^,^9^. In our present study, compared to patients with MCD, UCD patients exhibit significantly better survival, consistent with previous reports^2^, suggesting the involvement of a different etiology^9^. Based on these observations, we conducted further Cox regression analyses in our cohort of MCD patients. Moreover, considering that patients with concomitant autoimmune diseases and malignancies might have a different pathogenesis^9^, we excluded such patients in our Cox regression analysis.

The identification of prognostic factors for CD patients (especially for MCD patients, as UCD is associated with a more favorable outcome) is important for making clinical decisions. However, because of the low incidence of CD, few studies have investigated this issue. Indeed, most studies only used univariate analysis because of the limited number of patients^4^,^5^,^7^,^8^ and suggested that the MCD subtype, HIV status, complications (including renal complication), POEMS-related symptoms, extravascular fluid accumulation, and extent of disease involvement (one or both sides of the diaphragm) were possible prognostic factors. The findings of these previous studies were not often consistent and justified further large-scale studies. The only study to date that utilized multivariate Cox regression^6^ identified PNP as an independent risk factor. However, as mentioned above, that previous study enrolled a large proportion of PNP patients, which might not reflect the general population of CD patients. Herein, we conducted the largest retrospective study to date and utilized both univariate and multivariate Cox regression analyses for MCD patients. With univariate analysis, we identified several possible risk factors, some of which were consistent with prior studies (POEMS and renal involvement). With multivariate analysis, for the first time we identified reduced eGFR as an independent prognostic factor. Several possible factors could underlie this finding. First, renal complications have been identified as a possible risk factor in previous large-scale analyses^6^. Second, renal impairment has been suggested to be a prognostic factor in diseases with a similar etiology, such as multiple myeloma^15^ and POEMS syndrome^16^. Therefore, we suggest that close monitoring of renal function should be implemented for MCD patients in clinical practice.

Notably, despite the fact that this current study is of clinical importance, it also carries considerable limitations. First, as a retrospective study, it has an implicit bias for data collection and interpretation. Second, although the patient HIV status was known in this study, the status of another important virus (HHV-8) was unknown. HHV-8 has been recognized to play an important role in the pathogenesis of MCD^9^ and although not yet proven, it might impose some impact on patient prognoses according to the etiology of MCD. Third, the prognostic factor found in this study (renal function) could be influenced by diseases other than CD (hypertension, diabetes, etc.); fortunately, our CD patients were relatively younger and the incidences of these chronic diseases were low. Fourth, because of the rarity of MCD, there was no established standardized treatment protocol at our hospital (Appendix 1), so it was difficult to analyze the impact of different treatment strategies on the survival of our patients. Awareness of these limitations should aid in the design of future studies of MCD and ultimately benefit patients with this rare disease.

This largest study carried out to date was based on recently available data. Our findings indicate that UCD patients enjoy better survival than MCD patients and that renal function is an important prognostic factor in MCD patients.

## Additional Information

**How to cite this article**: Zhang, L. *et al.* Clinical spectrum and survival analysis of 145 cases of HIV-negative Castleman’s disease: renal function is an important prognostic factor. *Sci. Rep.* **6**, 23831; doi: 10.1038/srep23831 (2016).

## Supplementary Material

Supplementary Information

## Figures and Tables

**Table 1:** Survival analysis using Cox regression.

	Risk factors	Univariate analysis	Multivariate analysis		
		HR (95% CI)	P	HR (95% CI)	P
Demographic characteristics	Age at CD diagnosis (years)	1.03 (0.99–1.08)	0.15		
	**Gender, male (n)**	0.29 (0.08–1.06)	**0.06**	0.32 (0.09–1.16)	0.08
	HV variate (n)	0.90 (0.29–2.76)	0.85		
	The same side of the diaphram (n)	0.04 (0.00–42.18)	0.36		
	*Chronic diseases (n)	1.06 (0.29–3.85)	0.94		
	Hepatitis virus infection (n)	1.44 (0.19–11.08)	0.73		
	**Prior history of TB (n)**	4.51 (1.23–16.47)	**0.02**	2.66 (0.67–10.55)	0.16
	Development delay (n)	0.05 (0.00–8073.10)	0.62		
	**POEMS (n)**	3.03 (1.01–9.04)	**0.047**	2.01 (0.55–7.37)	0.29
	ECOG ≥ 1	25.98 (0.02–33441.67)	0.37		
Symptoms & signs	B symptom (n)	5.05 (0.65–38.98)	0.12		
	**Generalized symptoms (n)	1.00 (0.13–7.71)	1.00		
	Shortness of breath (n)	2.15 (0.72–6.43)	0.17		
	Rash (n)	1.97 (0.66–5.87)	0.22		
	Adenopathy (n)	2.79 (0.36–21.48)	0.33		
System involvement	***eGFR< 60 ml/min (n)	4.89 (1.63–14.73)	**0.005**	4.60 (1.50–14.12)	**0.008**
	Nephrotic syndrome (n)	0.05 (0.000–6256.44)	0.61		
	Pulmonary involvment (n)	1.51 (0.49–4.65)	0.47		
	Paraneoplastic pemphigus (n)	1.88 (0.24–14.68)	0.55		
Laboratory tests	WBC (10^9/L)	1.10 (0.94–1.29)	0.22		
	Hb (g/L)	1.01 (0.99–1.03)	0.21		
	Platelet (10^9/L)	1.00 (0.99–1.00)	0.90		
	Positive urine protein (n)	0.78 (0.23–3.05)	0.79		
	Positive fecal occult blood (n)	2.84 (0.36–22.32)	0.32		
	Serum albumin (g/L)	0.94 (0.87–1.02)	0.16		
	Tbil (umol/L)	0.97 (0.82–1.14)	0.68		
	ALP (U/L)	0.99 (0.98–1.01)	0.28		
	LDH (U/L)	1.00 (0.99–1.01)	0.81		
	hsCRP (mg/L)	0.99 (0.97–1.01)	0.19		
	ANA positive (n)	0.03 (0.00–24.59)	0.32		
	HbsAg positive (n)	1.82 (0.23–14.07)	0.57		
	HCV positive (n)	0.05 (0.00–7145.0)	0.80		
	EBV–DNA positive (n)	0.04 (0.00–12940.0)	0.75		
	CMV–DNA positive (n)	0.04 (0.00–12940.0)	0.75		
	M–protein positive (n)	2.08 (0.70–6.21)	0.19		

CD, Castleman’s disease; HV, hyaline–vascular; TB, tuberculosis; POEMS, polyneuropathy, organomegaly, endocrinopathy, monoclonal protein, skin changes; ECOG, Eastern Cooperative Oncology Group; WBC, white blood cell; Hb, hemoglobin; Tbil, total bilirubin; ALP, alkaline phosphatase; LDH, lactate dehydrogenase; hsCRP, hypersensitive c-reactive protein; ANA, anti-nuclear antibodies; HbsAg, hepatitis B surface antigen; HCV, hepatitis C; EBV, Epstein-Barr Virus; CMV, cytomegalovirus.

*Chronic disease: hypertension, diabetes, coronary heart disease, chronic liver disease, chronic kidney disease; **B symptom: fever, night sweats, weight loss; ***Generalized symptom: fatigue, malaise, appetite, pain; ****eGFR: estimated glomerular filtration rate with Modification of Diet in Renal Disease (MDRD) equation.

**Table 2:** Comparison studies which enrolled more than 100 CD patients.

	Dispenzieri A, *et al*.^5^	Dong Y, *et al*.^6^	Our study
Patient number	113	114	145
Median Age at CD diagnosis (years)	43	35	40
Male: Female	48 : 52	53.5 : 46.5	47.6 : 52.4
UCD: MCD	47 : 53	54.4 : 45.6	47.6 : 52.4
HV: PC: Mixed	47.8 : 47.8 : 4.4	59.6 : 26.3 : 14.1	51.0: 35.2 : 13.8
Paraneoplastic pemphigus (%)	1%	32.4%	4.8%
HIV status (positive cases/tested cases)	1/25	0/67	0/145
Years of patient enrollment	1948–2002	1977–2014	2000–2015
1–year Survival	Not mentioned	91.2%*	95.1%
2–year Survival	92.0%	Not mentioned	93.8%**
3–year Survival	Not mentioned	78.1%*	93.1%**
5–year Survival	76.0%	Not mentioned	91.0%
Risk factor for suvival***	Not mentioned	Paraneoplastic pemphigus	eGFR< 60 ml/min

CD, Castleman’s disease; UCD, unicentric CD; MCD, multicentric CD; HV, hyaline-vascular variant; PC, plasma cell variant; eGFR, estimated glomerular filtration rate with Modification of Diet in Renal Disease (MDRD) equation. *estimated from published data; **calculated from our database (data not reported in manuscript); ***with multivariate Cox regression.

## References

[b1] Castleman B., Iverson L. & Menendez V. P. Localized mediastinal lymphnode hyperplasia resembling thymoma. Cancer. 9, 822–830 (1956). 13356266 10.1002/1097-0142(195607/08)9:4<822::aid-cncr2820090430>3.0.co;2-4

[b2] Soumerai J. D., Sohani A. R. & Abramson J. S. Diagnosis and management of Castleman disease. Cancer control. 21, 266–278 (2014). 25310208 10.1177/107327481402100403

[b3] Talat N. & Schulte K. M. Castleman’s disease: systematic analysis of 416 patients from the literature. Oncologist. 16, 1316–1324 (2011). 21765191 10.1634/theoncologist.2011-0075 PMC3228165

[b4] Casper C. *et al.* Clinical characteristics and healthcare utilization of patients with multicentric Castleman disease. Br J Haematol. 168, 82–93 (2015). 25208471 10.1111/bjh.13111

[b5] Dispenzieri A. *et al.* The clinical spectrum of Castleman’s disease. Am J Hematol. 87, 997–1002 (2012). 22791417 10.1002/ajh.23291 PMC3900496

[b6] Dong Y. *et al.* Clinical and laboratory characterization of 114 cases of Castleman disease patients from a single centre: paraneoplastic pemphigus is an unfavourable prognostic factor. Br J Haematol. 169, 834–842 (2015). 25824806 10.1111/bjh.13378

[b7] Seo S. *et al.* Clinical features and outcomes in patients with human immunodeficiency virus-negative, multicentric Castleman’s disease: a single medical center experience. Blood Res. 49, 253–258 (2014). 25548759 10.5045/br.2014.49.4.253 PMC4278007

[b8] Kawabata H. *et al.* Clinical features and treatment of multicentric castleman’s disease: a retrospective study of 21 Japanese patients at a single institute. J Clin Exp Hematop. 53, 69–77 (2013). 23801137 10.3960/jslrt.53.69

[b9] Fajgenbaum D. C., van Rhee F. & Nabel C. S. HHV-8-negative, idiopathic multicentric Castleman disease: novel insights into biology, pathogenesis, and therapy. Blood. 123, 2924–2933 (2014). 24622327 10.1182/blood-2013-12-545087

[b10] Gill B. & Okie S. China and HIV - a window of opportunity. N Engl J Med. 356, 1801–1805 (2007). 17476005 10.1056/NEJMp078010

[b11] Levey A. S. *et al.* A more accurate method to estimate glomerular filtration rate from serum creatinine: a new prediction equation. Modification of Diet in Renal Disease Study Group. Ann Intern Med. 130, 461–470 (1999). 10075613 10.7326/0003-4819-130-6-199903160-00002

[b12] Li J. & Zhou D. B. New advances in the diagnosis and treatment of POEMS syndrome. Br J Haematol. 161, 303–315 (2013). 23398538 10.1111/bjh.12236

[b13] Wang J. *et al.* Paraneoplastic pemphigus associated with Castleman tumor: a commonly reported subtype of paraneoplastic pemphigus in China. Arch Dermatol. 141, 1285–1293 (2005). 16230567 10.1001/archderm.141.10.1285

[b14] Soulier J. *et al.* Kaposi’s sarcoma-associated herpesvirus-like DNA sequences in multicentric Castleman’s disease. Blood. 86, 1276–1280 (1995). 7632932

[b15] Kyle R. A. *et al.* Review of 1027 patients with newly diagnosed multiple myeloma. Mayo Clin Proc. 78, 21–33 (2003). 12528874 10.4065/78.1.21

[b16] Ye W. *et al.* Renal impairment in patients with polyneuropathy, organomegaly, endocrinopathy, monoclonal gammopathy and skin changes syndrome: incidence, treatment and outcome. Nephrol Dial Transplant., 10.1093/ndt/gfv261 (2015). 26130736

