# Supplementary material for: Clinical spectrum and survival analysis of 145 cases of HIV-negative Castleman’s disease: renal function is an important prognostic factor
Source: Sci Rep. 2016 Mar 31;6:23831. doi: 10.1038/srep23831 (PMC4814900; doi:10.1038/srep23831)
Supplement: Supplementary Information [file srep23831-s1.doc]

**Title：Clinical spectrum and survival analysis of 145 cases of HIV-negative Castleman’s disease: renal function is an important prognostic factor**

**Authors**: Lu Zhang ^1^, Zhiyuan Li ^2^, Xinxin Cao ^1^, Jun Feng ^1^, Dingrong Zhong ^2^, Shujie Wang ^1^, Daobin Zhou ^1^, Jian Li ^1 *^

| Appendix 1. Clinical characteristics of 145 CD patients | | |
| --- | --- | --- |
|  | Value | (%) |
| Gender |  |  |
| Male | 69 | 47.6 |
| Female | 76 | 52.4 |
| Clinical subtype |  |  |
| Unicentric CD | 69 | 47.6 |
| Multicentric CD | 76 | 52.4 |
| Pathological variate |  |  |
| HV variate | 74 | 51.0 |
| PC variate | 51 | 35.2 |
| Mixed | 20 | 13.8 |
| The same side of the diaphram | 79 | 54.5 |
| POEMS | 19 | 13.1 |
| Paraneoplastic pemphigus | 7 | 4.8 |
| eGFR |  |  |
| <60ml/min | 17 | 11.7 |
| ≥60ml/min | 128 | 88.3 |
| *Treatment |  |  |
| Biopsy of the periperal lymph node only | 7 | 4.8 |
| Surgery | 80 | 55.2 |
| Steroids±IVIG±Immunosuppressive therapy | 13 | 9.0 |
| **CHOP-like chemotherapy±Rituximab | 47 | 32.4 |
| ***High-dose dexamethasone based therapy | 7 | 4.8 |
| ASCT | 8 | 5.5 |
| CD, Castleman's disease; HV, hyaline-vascular; PC, plasma cell; POEMS, polyneuropathy, organomegaly, endocrinopathy, monoclonal protein, skin changes; eGFR: estimated glomerular filtration rate with Modification of Diet in Renal Disease (MDRD) equation;IVIG,intravenous immunoglobulin; CHOP, cyclophosphamide, doxorubicin, vincristine, and prednisone; ASCT, autologous stem cell transplantation | | |
| *Treatment strategies overlap for some patients; **8 patients received rituximab; ***Including Melphalan+Dexamethasone (n=3), Lenalidomide+Dexamethasone (n=3)，Thalidomide+Cyclophosphamide+Dexamethasone (n=1) | | |

| **Appendix 2. Comparison of risk factor distribution between survivors and non-survivors** | | | | | | | | |
| --- | --- | --- | --- | --- | --- | --- | --- | --- |
|  |  |  | Non-survivors | | Survivors | | |  |
|  |  | General Characteristics | Value | No | Value | No | P |  |
|  | Demographic characteristics | Age at CD diagnosis (years) | 45.3± 13.4 | 13 | 41.5 ± 14.6 | 63 | 0.38 |  |
|  |  | **Gender, male (n)** | 3 | 13 | 37 | 63 | **0.03** |  |
|  |  | HV variate (n) | 5 | 13 | 23 | 63 | 0.89 |  |
|  |  | The same side of the diaphram (n) | 0 | 13 | 10 | 63 | 0.12 |  |
|  |  | Chronic diseases (n)* | 3 | 13 | 17 | 63 | 0.77 |  |
|  |  | Hepatitis virus infection (n) | 1 | 13 | 4 | 63 | 0.86 |  |
|  |  | **Prior history of TB (n)** | 3 | 13 | 3 | 63 | **0.03** |  |
|  |  | Development delay (n) | 0 | 13 | 3 | 63 | 0.44 |  |
|  |  | **POEMS (n)** | 6 | 13 | 12 | 63 | **0.04** |  |
|  |  | ECOG≥1 (n) | 13 | 13 | 51 | 63 | 0.09 |  |
|  | Symptoms & signs | **B symptom (n) | 12 | 13 | 44 | 63 | 0.09 |  |
|  |  | ***Generalized symptoms (n) | 12 | 13 | 57 | 63 | 0.84 |  |
|  |  | Shortness of breath (n) | 7 | 13 | 21 | 63 | 0.16 |  |
|  |  | Rash (n) | 6 | 13 | 18 | 63 | 0.21 |  |
|  |  | Adenopathy (n) | 12 | 13 | 52 | 63 | 0.38 |  |
|  | System involvement | ******eGFR<60ml/min (n)** | 6 | 13 | 9 | 63 | **0.009** |  |
|  |  | Nephrotic syndrome (n) | 0 | 13 | 3 | 63 | 0.42 |  |
|  |  | Pulmonary involvment (n) | 5 | 13 | 16 | 63 | 0.34 |  |
|  |  | Paraneoplastic pemphigus (n) | 1 | 13 | 1 | 63 | 0.21 |  |
|  | Laboratory tests | WBC (10^9/L) | 7.9± 3.5 | 13 | 6.9± 2.8 | 63 | 0.28 |  |
|  |  | Hb (g/L) | 120.1 ± 21.9 | 13 | 108.1 ± 29.4 | 63 | 0.17 |  |
|  |  | Platelet (10^9/L) | 310.2 ± 166.2 | 13 | 318.4± 186.2 | 63 | 0.88 |  |
|  |  | Positive urine protein (n) | 3 | 13 | 19 | 63 | 0.61 |  |
|  |  | Positive fecal occult blood (n) | 1 | 13 | 2 | 63 | 0.45 |  |
|  |  | Serum albumin (g/L) | 30.7 ± 4.0 | 13 | 33.0 ± 7.0 | 63 | 0.11 |  |
|  |  | Tbil (umol/L) | 7.89 ± 4.17 | 13 | 8.66± 4.25 | 63 | 0.55 |  |
|  |  | ALP (U/L) | 68.8 ± 20.1 | 13 | 107.4± 118.3 | 63 | 0.25 |  |
|  |  | LDH (U/L) | 182.2 ± 171.5 | 13 | 146.4± 149.4 | 63 | 0.45 |  |
|  |  | hsCRP (mg/L) | 13.6 ± 14.6 | 12 | 45.4± 65.6 | 54 | 0.10 |  |
|  |  | ANA positive (n) | 0 | 10 | 12 | 54 | 0.10 |  |
|  |  | HbsAg positive (n) | 1 | 13 | 3 | 63 | 0.67 |  |
|  |  | HCV positive (n) | 0 | 13 | 1 | 63 | 0.65 |  |
|  |  | EBV-DNA positive (n) | 0 | 3 | 1 | 29 | 0.74 |  |
|  |  | CMV-DNA positive (n) | 0 | 3 | 1 | 29 | 0.74 |  |
|  |  | M-protein positive (n) | 6 | 13 | 14 | 63 | 0.07 |  |
|  | Treatment | Chemotherapy (n) | 9 | 13 | 45 | 63 | 0.87 |  |
|  | CD, Castleman's disease; HV, hyaline-vascular; TB, tuberculosis; POEMS, polyneuropathy, organomegaly, endocrinopathy, monoclonal protein, skin changes; ECOG, Eastern Cooperative Oncology Group; WBC, white blood cell; Hb, hemoglobin; Tbil, total bilirubin; ALP, alkaline phosphatase; LDH, lactate dehydrogenase; hsCRP, hypersensitive c-reactive protein; ANA, anti-nuclear antibodies; HbsAg, hepatitis B surface antigen; HCV, hepatitis C; EBV, Epstein-Barr Virus; CMV, cytomegalovirus; | | | | | | |  |
|  | *Chronic disease: hypertension, diabetes, coronary heart disease, chronic liver disease, chronic kidney disease; **B symptom: fever, night sweats, weight loss; ***Generalized symptom: fatigue, malaise, appetite, pain; ****eGFR: estimated glomerular filtration rate with Modification of Diet in Renal Disease (MDRD) equation. | | | | | | |  |
